# Comparing the Efficacy of Manual Therapy and Exercise to Synchronized Telerehabilitation with Self-Manual Therapy and Exercise in Treating Subacromial Pain Syndrome: A Randomized Controlled Trial

**DOI:** 10.3390/healthcare12111074

**Published:** 2024-05-24

**Authors:** Erman Berk Çelik, Aysenur Tuncer

**Affiliations:** 1 Department of Physiotherapy and Rehabilitation, Institute of Health Sciences, Mardin Artuklu University, 47200 Mardin, Türkiye; 2 Department of Physiotherapy and Rehabilitation, Institute of Health Sciences, Hasan Kalyoncu University, 27410 Gaziantep, Türkiye; aysenur.tuncer@hku.edu.tr

**Keywords:** subacromial pain syndrome, manual therapy, telerehabilitation

## Abstract

This study aimed to investigate the efficacy of manual therapy and exercise versus synchronized telerehabilitation with self-manual therapy and exercise in treating Subacromial Pain Syndrome (SAPS). Sixty individuals diagnosed with SPS, aged 18–50 years, were randomly assigned to home exercise (HE), manual therapy (MT), and telerehabilitation (TR) groups. Treatment protocols were administered over 8 weeks and included specific exercises and therapy interventions. Outcome measures included the Visual Pain Scale (VAS), shoulder range of motion (ROM) via goniometric measurements, Quick Disability Arm-Shoulder-Hand Problems Survey (Q-DASH), and patient satisfaction. Results revealed that both MT and TR groups exhibited reduced pain, increased ROM, lower Q-DASH scores, and higher patient satisfaction than the HE group. However, no significant differences were found between the MT and TR groups regarding pain levels, ROM, Q-DASH scores, or patient satisfaction. The study concludes that both telerehabilitation and manual therapy effectively alleviate pain and are well-received by patients with SPS. Additionally, manual therapy demonstrates superiority in enhancing functional levels compared to exercise-based interventions (Registration: NCT05200130).

## 1. Introduction

Shoulder pain is a common musculoskeletal disease [1,2]. Compared to other musculoskeletal diseases, it is the third most common musculoskeletal problem [2]. A total of 48% of these patients are diagnosed with Subacromial Pain Syndrome (SAPS). This is a chronic complaint, with 54% of patients reporting persistent symptoms even after 3 years [3].

SAPS is a broad term that encompasses various shoulder conditions causing pain. The pain is usually one-sided and worsens during or after arm-raising activities. It is localized around the acromion, a bony prominence at the top of the shoulder blade. SAPS is used to describe pain arising from any lesion within the structures in the subacromial space, the area beneath the acromion. This includes conditions like rotator cuff tendinitis, calcific tendinitis, bursitis, partial or complete tears, and cuff degeneration [4].

Exercise has an important place in SAPS treatment. Although a standard protocol of exercise therapy has not been established, exercise can decrease pain and improve shoulder function [5,6,7]. Exercise programs should focus on reducing pain, correcting muscle imbalances, and increasing strength and neuromuscular control. The treatment program should encompass the rehabilitation of all structures affected by SAPS [8].

The goals of treatment are to halt the inflammatory process, reduce pain, achieve a normal, functional, and stable joint, maintain normal joint movement, and prevent the occurrence of progressive degenerative changes. Conservative treatment in the management of SAPS involves reducing pain and inflammation of the joint and soft tissues, correcting mobility impairments, improving scapulohumeral rhythm, re-educating posture and movements, increasing strength and endurance, providing functional training for daily activities, making ergonomic modifications, and patient education [9].

The aim before initiating an exercise program is to reduce inflammatory reactions and pain to ensure full participation in exercises. For this purpose, non-steroidal anti-inflammatory drug therapy, ice application, corticosteroid injections, manual therapy (MT) techniques, and dry needling, as well as electrotherapy agents such as Transcutaneous Electrical Nerve Stimulation (TENS) and Ultrasound (US), are used [10,11].

The effectiveness of manual treatment for patients with SAPS is a frequently investigated topic [12]. Manual treatment techniques are thought to reduce pain thanks to their biomechanical and neurophysiological effects [13]. The purpose of manual therapy, including joint mobilization, is to alleviate pain and restore function. Manual therapy is applied to the joints and related soft tissues at varying speeds and amplitudes. Manual therapy aims to stimulate peripheral mechanoreceptors, inhibit nociceptors, and increase synovial nutrition, ultimately leading to pain reduction. Mobilizations are classified based on intensity to produce beneficial effects such as the realignment of collagen, increased fiber glide, and breakage of adhesions to restore normal joint mobility [14]. 

Manual therapy encompasses a range of hands-on techniques applied by a practitioner in a clinical setting. These techniques may involve passive interventions where the patient remains passive while the therapist manipulates or mobilizes their joints, soft tissues, or muscles [15]. Additionally, manual therapy may include self-mobilization where the patient participates actively in the movement or manipulation process, guided by the therapist’s instructions [16].

Self-mobilization techniques can involve various movements, stretches, or manipulations aimed at specific joints or areas of the body. They are used as part of a comprehensive treatment plan for conditions such as joint stiffness, muscle tightness, or limited range of motion [17].

Telerehabilitation is a popular approach that stands out with the effects of the pandemic process we live in [18]. Telerehabilitation is the delivery of rehabilitation services remotely using telecommunication technologies. It can be delivered in real-time or through recorded videos, using phones and video conferencing platforms, or in virtual reality [19]. Synchronized modality is the term used to describe telerehabilitation that co-occurs in real-time, with data, speech, and information transfer (e.g., video conferencing). Asynchronized modality, on the other hand, involves stored or transmitted digital images [20]. With these remote rehabilitation services, healthcare professionals can evaluate patients, monitor them, and respond to the treatment program [21]. 

It has been suggested that telerehabilitation can be used to assess range of motion, muscle strength, balance, gait, and several functional outcome measures in physical therapy and rehabilitation [22]. To describe and demonstrate musculoskeletal and neurological examination techniques that can be effectively used in telerehabilitation, 20 experts in the field have published a set of diagnostic tests that can be used to examine the neck, shoulder, elbow, hand, and wrist, along with a range of motion, muscle strength, and observational examination guide, based on their ease of instructing patients correctly and their ability to adapt to safely perform the movements in their environment without compromising validity [23]. However, low to moderate validity has been reported for various specialized orthopedic tests and neurodynamic tests. Diagnostic concordance compared to face-to-face evaluations has been assessed and found to have moderate validity [24]. 

In the literature, there was no study comparing the method of manual therapy with exercise and synchronized telerehabilitation with manual therapy applied to exercise in cases with SAPS and manual treatments that can be applied to exercise with additional self-mobilization. This study aims to compare the efficacy of two different treatment approaches for SAPS, manual therapy and exercise versus synchronized telerehabilitation with self-mobilization and exercise. Additionally, a home exercise program is included to evaluate the effectiveness of these treatment methods as a control group. The study investigates which treatment method is more effective in alleviating pain, improving shoulder range of motion, reducing disability, and patient satisfaction in individuals diagnosed with SAPS. By comparing the outcomes of these two treatment approaches with the control group, the study aims to provide insights into the effectiveness of telerehabilitation compared to manual therapy and exercise interventions for SAPS management.

The hypothesis anticipates that both manual therapy and telerehabilitation interventions will showcase comparable efficacy in alleviating pain and enhancing shoulder function. Furthermore, the study seeks to explore the level of patient satisfaction associated with these interventions.

## 2. Materials and Methods

### 2.1. Ethical Approval of the Study Protocol

The study protocol received approval from the Hasan Kalyoncu University, Faculty of Medicine Ethics Committee on 21 June 2021, with the reference number 2021/074. The study adhered to the principles of the Declaration of Helsinki.

### 2.2. Participants

A power analysis using OpeneEpi version 3 determined that a minimum of 26 subjects per group was necessary, considering α = 0.05 and 1 − β = 0.80 [25]. Before the research, volunteers were informed about the treatment and evaluation protocol. Those willing to continue signed a volunteer form.

#### 2.2.1. Inclusion Criteria

Individuals aged 18–50 years with complaints of shoulder pain were included in this study. The presence of shoulder pain lasting more than 6 weeks that restricts activity was also an inclusion criterion. Informed consent provided by patients who had been briefed about the study and had given written consent to participate was mandatory.

#### 2.2.2. Exclusion Criteria

Individuals with other orthopedic, neurological, or systemic problems affecting the neck–shoulder–back complex were excluded from the study. Patients with heart failure or using a pacemaker were also excluded. Degenerative joint disease of the shoulder joint complex and a diagnosis of a frozen shoulder were exclusion criteria. A body mass index (BMI) > 30 kg/m^2^ was another criterion for exclusion. Participation in a physical therapy program for the same shoulder joint in the past year and steroid injections administered in the past 6 months were also exclusion criteria. Finally, the use of non-steroidal anti-inflammatory drugs is grounds for exclusion. A total of 78 individuals were included initially, with 60 participants completing the study after exclusions. 

### 2.3. Clinical Tests

Research has suggested the utilization of the Hawkins–Kennedy Test, Painful Arc Test (Abduction/Flexion at 60–120 degrees), and Infraspinatus Resistance Test [4]. In our study, inclusion criteria required patients to have at least two of these tests be positive [26]. The Drop-Arm Sign test was also used as an exclusion criterion for supraspinatus tears. If this test was positive, the patient was excluded from the study [4]. These tests were conducted by an experienced physician before, after 8 weeks of treatment, and at the 12-week follow-up.

### 2.4. Randomization

We performed the study using the sealed envelope method. Envelopes were prepared that had been assigned to each group, and participants randomly selected an envelope to be allocated to the home exercise, manual therapy, or telerehabilitation group. A double-blind design was not used. Due to the nature of the intervention (e.g., manual therapy) in this study, it was not possible to prevent researchers and participants from knowing which group they were assigned to.

### 2.5. Treatments

Exercise Program: In our study, exercises for rotator cuff strengthening and scapular stabilizing were utilized, along with shoulder capsular stretching techniques. It was recommended that strengthening exercises should be pain-free. We used the same exercise rehabilitation protocol across all study groups. The exercise program lasts approximately 45 min.

Resistance bands of low, medium, and high levels were used for muscle-strengthening exercises. In selecting the resistance band, the one that the patient could use to bring their arm to 90° abduction and hold for 3 s without losing stability or experiencing pain was chosen. The resistance was increased after 4 weeks [27]. 

Muscle Strengthening (3 days): The aim was to improve strength and stability in the rotator cuff and scapular muscles, which can help alleviate impingement symptoms. This comprised 3 sets of 10 repetitions with control of the release, 3 sets of 15 reps in the second week, and 3 sets of 20 reps in the third week. There was a 60 s rest between sets for strengthening exercises [28].

#### 2.5.1. Rotator Cuff Strengthening

The participant grasped the band with enough tension to feel a slight resistance. Then, the participant held the position for a moment at the top, feeling the contraction in the muscles, then slowly returned to the starting position, maintaining tension in the band throughout.

1. Shoulder internal rotation without scapular humeral abduction:

The participant attached the elastic resistance band to the fixed object, ensuring that the fixed point remained laterally outside of the body. Then, the participant maintained a neutral arm position with elbows bent at 90 degrees and palms facing the body. The upper arms should be close to the sides of the body, not fully pressed against them. They were then instructed to initiate an internal rotation of the shoulders. Subscapularis, deltoid, and teres major.

2. Shoulder external rotation without scapular humeral abduction:

The participant attached the elastic resistance band to the fixed object, ensuring that the fixed point remained medially inside of the body. They maintained a neutral arm position with elbows bent at 90 degrees and palms facing the body. In this position, the upper arms should be close to the sides of the body, not fully pressed against them. They were then instructed to initiate an external rotation of the shoulders. Infraspinatus and teres minor.

3. Full-Can Exercise:

The exercise began by securing the resistance band under the foot and holding the opposite end. The hands were raised in front of the body to approximately shoulder height, with thumbs pointing up and elbows held in a slightly bent position. The arms formed an angle of approximately 30 degrees with the sides of the body. Instructions were given to raise the arms up to shoulder height. Supraspinatus.

#### 2.5.2. Scapular Stabilization Strengthening

1. Row: This began by securely anchoring the resistance band to a stable point level with the shoulder. Standing with the feet shoulder-width apart and holding one end of the band in each hand, with palms facing each other, the arms were extended straight out in front at shoulder height. With the elbows slightly bent, the band was pulled towards the torso, squeezing the scapulas. Middle trapezius and deltoid.
2. Chair press: Seated on a chair or table, place both hands firmly on the sides of the chair, palm down and fingers pointed outward. The hands should be placed underneath the shoulders. Slowly push downward through the hands to elevate the body. Low trapezius.
3. Scapular adduction with shoulder extension.

This began by securely anchoring the band to a stable point in front of the body. Standing with feet shoulder-width apart and holding one end of the band in each hand, palms facing each other. The arms should be in a neutral position, elbows straight. Pull the band extending it away from the body. Latisimus dorsi and deltoid.

Shoulder Stretching (5 days): To improve flexibility in the shoulder capsule and surrounding muscles, which can contribute to impingement. Each stretch is held for 30 s and repeated 5 times, with a 10 s rest between each stretch.

1. Anterior shoulder stretch: They performed this stretch by placing their hands at shoulder level on each side of a door or in a corner of a room, then leaning forward into the door or corner and holding the position. Pectoralis major.
2. Posterior shoulder stretch: Participants were instructed to bring their involved arm across the front of their body. They were asked to hold the elbow with their other arm and gently flex the bent arm, allowing it to pull the other arm across the chest until they felt a stretch in the back of the shoulder. Deltoid.
3. Wand stretching exercise: Shoulder flexion overhead reach, the patient stood with their feet shoulder-width apart. They held the wand with both hands, palms facing down, hands shoulder-width apart. They slowly lifted the wand overhead as far as was comfortable, keeping their arms straight. They held the wand overhead, feeling a stretch in their shoulders and upper back. Shoulder flexion lateral raise (both sides). The patient stood with their feet shoulder-width apart. They held the wand in front of their thighs with both hands, palms facing down, hands shoulder-width apart. They slowly raised the wand out to the sides until their arms were parallel to the floor. They held the position briefly, ensuring their arms were straight and parallel to the ground. Then, they lowered the wand back to the starting position. Shoulder external rotation swing (side to side). The patient stood with their feet shoulder-width apart. They held the wand with both hands, palms facing up, elbows bent at 90 degrees, and elbows close to their sides. Keeping their elbows at their sides, they rotated the wand outward to the right, allowing their shoulders to externally rotate. Then, they rotated the wand outward to the left. They swung the wand gently from side to side, maintaining control and a comfortable range of motion.

#### 2.5.3. Home Exercise Group (HE)

This group received recommendations related to daily life activities. These suggestions included avoiding excessively strenuous and repetitive activities and not sleeping on the painful side.

- The physiotherapist monitored exercise continuity through weekly reminders.
- Patients documented their exercise follow-up for 8 weeks.

#### 2.5.4. Manual Therapy Group

Orthopedic manual physical therapy glenohumeral gliding techniques according to Christopher H. Wise were used in grades II and III [29]. For each mobilization, 5 reps were applied, consisting of 10 s of holding and relaxing. Each session took 10 min. 

#### 2.5.5. Mobilizations

Glenohumeral Inferior Glide: The patient can be lying on their back or sitting upright. The clinician sits or stands on the same side as the mobilized shoulder, facing towards the head. The clinician places their stabilizing hand wrapped in a towel within the patient’s axillary place to support the shoulder. The mobilization hand then exerts an inferior glide by moving the humeral head downwards.

Glenohumeral Posterior Glide: The patient lies on their back. The clinician stands on the same side as the mobilized shoulder, facing towards the head. The clinician places their mobilization hand on the anterior aspect of the humeral head and guides the shoulder into external rotation using the stabilizing hand. The mobilization hand applies a postero-lateral glide as the shoulder is moved into external rotation.

Glenohumeral Anterior Glide: The patient lies face down. The clinician stands on the same side as the mobilized shoulder, facing towards the head. The clinician places their forearm over the scapula to stabilize it and move it into a neutral position. The mobilization hand then makes contact with the posterior aspect of the humeral head and applies an anterior glide by moving it forward.

- Advised on daily life activities, receiving the same home exercise program.
- Instructed to perform exercises five days a week.
- An additional 8 weeks of exercise including joint mobilizations 2 days a week.

#### 2.5.6. Telerehabilitation Group

Before the patients actively performed self-mobilization exercises, they underwent training. They were taught to apply the same intensity of mobilization on themselves as the physiotherapist applied to them. Each patient was instructed on how and where to apply the force through practical exercises. They were then asked to perform these exercises at home actively. Each mobilization application involved approximately 10 s of holding and relaxing. Each mobilization application involved approximately 5 reps comprising 10 s of holding and relaxing. Each session for mobilizations takes 10 min.

#### 2.5.7. Self-Mobilizations

Glenohumeral Self-Inferior Glide: Mobilization is intended for the arm, fixed in abduction on the lateral side of the body while seated, with the unaffected hand actively sliding the humeral head inferiorly, feeling “gapping”. 

Glenohumeral Self-Anterior Glide: Mobilization is intended for the arm, supported on the posterior side of the body on a table in extension while seated, with the unaffected hand actively sliding the humeral head anteriorly, feeling “gapping”. 

Glenohumeral Self-Posterior Glide: Mobilization is intended for the arm, supported in lateral and external rotation on a table while seated, with the unaffected hand actively sliding the humeral head posteriorly, feeling “gapping”.

- Advised about daily life activities and learned self-mobilization techniques with the same home exercise program.
- Instructed to perform exercises five days a week and two days a week for 45 min via video conferencing with a physiotherapist.
- Exercises and self-mobilization methods were performed against the physiotherapist under the home exercise program.

### 2.6. Outcomes Measures 

#### 2.6.1. Visual Pain Scale (VAS)

In our study, the VAS 0–10 cm form was used to assess pain. This form consists of a straight line spanning 10 cm. On the left end of this line, 0 indicates “no pain” while 10 signifies “unbearable pain” [30]. It is one of the best methods to subjectively evaluate the patient’s pain at that moment [31]. Patients were instructed to indicate the intensity of pain experienced during both activity and nighttime rest over the previous 24 h on this form. Marking was measured with the help of a ruler [32].

#### 2.6.2. Range of Motion (ROM)

Goniometric measurements were made for shoulder flexion, abduction, and internal and external rotation movements to assess the range of shoulder joint movement. Measurements were recorded in degrees using the universal goniometer [33]. 

#### 2.6.3. Quick Disability Arm-Shoulder-Hand Problems Survey (Q-DASH)

Q-DASH is a questionnaire filled out by patients with upper limb problems to determine pain and functionality levels during daily life activities. Q-DASH consists of 11 questions. In the Q-DASH survey, scoring for each section is between 0–100 (0: no disability, 100: most serious disability). Answers are answered between one and five (1: no difficulty, 2: mild difficulty, 3: medium difficulty, 4: extreme difficulty, 5: never being able to) [34].

#### 2.6.4. Patient Satisfaction Assessment

Patient satisfaction with the treatment method received was evaluated using the VAS form in the 12th-week measurements. Patients will be asked to give a score of VAS 0–10 cm, indicating satisfaction with using their shoulder in daily life activities compared to before treatment. A high score shows satisfaction with treatment.

### 2.7. Statistical Analysis

The descriptive statistics for continuous variables were presented as means and standard deviations (X ± SD). The normal distribution of the data was assessed using the Kolmogorov–Smirnov test. In comparing the Home Exercise, Manual Therapy, and Telerehabilitation groups, the Kruskal–Wallis test was utilized. To identify differences between different groups, the Mann–Whitney U test was employed. For comparing measurements taken before treatment, at 8 weeks, and at 12 weeks within each of the three groups, the Friedman test was conducted. The Wilcoxon signed-rank test was used to determine which group contributed to any observed differences. A significance level of *p* < 0.05 was considered for critical decision-making in the study. Data analysis was performed using SPSS 25.0 (Statistical Packages for Social Sciences) software.

## 3. Results

### 3.1. Patients 

A total of 60 people, including 20 people, were included in each of the Home Exercise (HE), Manual Therapy (MT), and Telerehabilitation (TR) groups. The average age was 39.75 ± 10.36 years in the HE group, 24.27 ± 2.4 kg/m^2^ in the TR group, and 41.2 ± 8.08 years in the MT group. No significant difference was found between the female and male ratios of MT and Telerehabilitation group patients (Table 1).

### 3.2. Pain Assessment 

The bilateral comparison was similar in the MT and TR groups (*p* > 0.05). The pain level in the MT and TR groups was better than in the HE group (*p* < 0.05) (Table 2). 

### 3.3. Functional Assessment

#### Range of Motion (ROM)

When looking at the bilateral comparison of shoulder ROM, increases in ROM in the manual treatment and telerehabilitation groups were found to be higher than in the home exercise group (*p* < 0.05). ROM scores in the manual treatment and telerehabilitation groups increased at a similar level (*p* > 0.05) (Table 3).

In the bilateral comparison of the groups, Q-DASH levels were found to be low in the MT group at 8 and 12 weeks compared to the HE and TR groups (*p* < 0.05). Compared to the HE and TR groups, the groups were not superior to each other (*p* > 0.05) (Table 4).

### 3.4. Patient Satisfaction Assessment

When looking at Patient Satisfaction VAS values, patients in the manual treatment and telerehabilitation groups had higher levels of treatment satisfaction than in the home exercise group (*p* < 0.05) (Table 5).

## 4. Discussion

Based on the findings of our study, we aimed to evaluate the comparative effectiveness of different treatment modalities, including a home exercise program, manual therapy plus exercise, and self-mobilization plus exercise telerehabilitation supported by video call. Our results revealed discernible differences among the groups concerning pain, functionality, and patient satisfaction levels.

Initially, we recorded consistent demographic information for all patient groups, indicating similar age, height, and body mass index distributions. Notably, our study determined an average age of 40 across the groups, with no significant correlation found between age and pain severity.

Several studies underscore the critical role of exercise in treating subacromial pain syndrome. While the effectiveness of manual therapy remains a topic of interest, a systematic review indicated that exercise programs alone could yield comparable results to those combined with manual therapy [35]. These findings align with existing guidelines that endorse exercise prescriptions for SAPS and recommend the incorporation of various adjunct therapies, including manual techniques and psychosocial interventions [36].

Manual therapy, known for its effects on muscles, joints, soft tissues, and the neurovascular system, has demonstrated promise in reducing pain, as supported by both our study and the existing literature [12]. However, challenges in evaluating the precise efficacy of manual therapy interventions in SAPS persist due to insufficient clarity on the intervention differences and application methods within various studies [37].

Our study further supports the importance of self-mobilization approaches, which have been observed to be beneficial in various musculoskeletal conditions. Notably, we found that both manual therapy and telerehabilitation-supported self-mobilization methods were effective in managing pain, with home exercise intervals demonstrating higher pain levels compared to the intervention groups.

Self-mobilization is a type of mobilization that patients apply to themselves and can involve the use of equipment [15]. Studies conducted on the self-mobilization approach in the literature were found to be present in subacromial pain syndrome, increasing thoracic mobilization, treating ankle injuries, equipment-assisted hamstring flexibility enhancement, sacroiliac dysfunction, and neck pain [38].

Additionally, we observed significant improvements in joint range of motion across all three groups, consistent with the existing literature. Both manual therapy interventions and telerehabilitation-supported self-mobilizations were found to relax tightened tissues around the shoulder joint and contribute to muscle strengthening, ultimately facilitating recovery.

In contrast, Park et al., in their randomized controlled study, did not observe a significant difference in joint range of motion measurements between the group receiving additional mobilization for subacromial pain syndrome and the group included in the specific exercise program [39].

Telerehabilitation, the primary focus of our study, has been extensively researched in evaluating and implementing treatment programs for various medical conditions. Unlike previous research, our study offers valuable insights into the comparative effectiveness of clinical manual therapy versus telerehabilitation-assisted self-mobilization. Furthermore, the use of telerehabilitation methods has garnered attention due to its potential to reduce treatment costs, save time, and improve treatment accessibility, keeping pace with advancing technological developments [40]. Our findings indicate that the telerehabilitation group exhibited pain levels and joint range of motion measurements similar to the manual therapy group. However, the manual therapy group demonstrated the highest increase in functionality and Q-DASH scores, indicating superior functionality improvement compared to the other groups.

Telerehabilitation is used in the assessment and implementation of treatment programs for musculoskeletal diseases, cardio-pulmonary rehabilitation, neurological conditions, and geriatric and pediatric patients [41,42,43,44,45]. In the literature, no study comparing clinical manual therapy with telerehabilitation-supported self-mobilization, as in our study, has been found.

Greiner et al. reported that in their study involving 132 participants who underwent shoulder surgery and received a telerehabilitation-supported exercise program, the majority of patients experienced improvement and were satisfied with this treatment [46]. In a study examining the satisfaction levels of both the practitioner and the patient in telerehabilitation programs applied to musculoskeletal disorders, both the practitioner and the patient reported satisfaction with this application. Patients recommended the combination of telerehabilitation with face-to-face rehabilitation applications [41].

In addition, in the VAS satisfaction assessment that we used to examine the satisfaction levels of our patients, it was determined that the satisfaction levels of the TR group and the MT group were similar, but the satisfaction levels of the HE group were lower than these two groups.

While our study has contributed valuable insights, it is crucial to note certain limitations. Specifically, we acknowledge the need for a more comprehensive assessment of patient satisfaction, including evaluating technological ease-of-use, patient preferences for telerehabilitation over face-to-face treatment, and the recording of technical and infrastructure issues experienced by patients. We believe that these detailed records could significantly inform future studies in this domain.

Lastly, the comparison between face-to-face and online measurements of the telerehabilitation group was not conducted in our study. This comparison could have provided valuable insights into the feasibility and reliability of online assessments and measurements, thereby enhancing our understanding of the applicability of telerehabilitation in clinical settings.

## 5. Conclusions

In the study, it was found that both manual therapy and telerehabilitation groups exhibited similar pain levels, while the group performing home exercises showed a lesser reduction in pain intensity. The research highlighted a significant increase in shoulder joint range of motion in individuals with SAPS, with manual therapy and telerehabilitation surpassing the home exercise group’s improvement. Moreover, both manual therapy and telerehabilitation interventions were seen to enhance functionality in patients with subacromial pain syndrome, with the most noticeable improvement observed in the manual therapy group. The study indicated that satisfaction levels were comparable between the manual therapy and telerehabilitation groups, surpassing those in the home exercise group. Telerehabilitation-supported exercise and self-mobilizations were deemed as effective as face-to-face exercises and manual therapy, reducing patient pain and improving functionality.

Exercise interventions play a crucial role in managing subacromial impingement syndrome. Therefore, we suggest designing patients’ rehabilitation programs with a strong emphasis on exercise-based regimens. During extraordinary circumstances like a pandemic, when factors such as transportation may hinder traditional treatment approaches, we advocate the utilization of telerehabilitation-supported treatment methods in situations where face-to-face treatment options are limited. We believe that telerehabilitation practices facilitate enhanced patient access to treatment. For patients in the telerehabilitation group, we propose the integration of self-mobilization techniques, known as self-mobilization, into the treatment plan. These techniques are designed for easy application by the patients themselves. Our observations indicate that both manual therapy and telerehabilitation-supported self-mobilization interventions significantly impact patient treatment satisfaction. Therefore, we recommend maintaining continuous patient interaction throughout the treatment process, even in an online setting.

## Figures and Tables

**Table 1:** Comparison of patients’ socio-demographic data.

	Home Exercise Group (n = 20)	Manual Therapy Group (n = 20)	Telerehabilitation Group (n = 20)	H	*p* ^1^
Age (year)	39.75 ± 10.36	41.2 ± 8.08	38.3 ± 7.67	3.423	0.622
				**ꭓ^2^**	***p* ^2^**
Gender					
Woman	8 (40.0%)	11 (55.0%)	12 (60.0%)	1.322	0.448
Man	12 (60.0%)	9 (45.0%)	8 (40.0%)		

*p* < 0.05, *p*^1^: Kruskal–Wallis Test, *p*^2^: Chi-Square Test.

**Table 2:** Pain levels.

Pain		Home Exercise Group			Manual Therapy Group	Tele Rehabilitation Group	Between Groups					
							HE-MT		HE-TR		MT-TR	
		X ± SD			X ± SD	X ± SD	z	*p* ^2^	z	*p* ^2^	z	*p* ^2^
VAS Activity	Before Treatment	7.94 ± 0.68	8.28 ± 0.38	7.83 ± 0.54								
	After Treatment 8th week	6.68 ± 0.76			5.12 ± 0.75	5.57 ± 0.66	2.42	<0.001 *	2.49	<0.001 *	1.65	0.112
	Follow-up 12th week	5.79 ± 1.05			3.42 ± 0.71	4.34 ± 0.64	2.85	<0.001 *	2.93	<0.001 *	1.12	0.281
	*p* ^1^	0.032 *	<0.001 *	0.021 *								
VAS Night	Before Treatment	7.06 ± 0.64	7.79 ± 0.51	6.92 ± 0.49								
	After Treatment 8th week	6.68 ± 0.76			5.12 ± 0.75	5.57 ± 0.66	2.88	<0.001 *	2.58	<0.001 *	1.63	0.118
	Follow-up 12th week	5.07 ± 0.82			3.39 ± 0.66	4.17 ± 0.73	2.74	<0.001 *	2.76	<0.001 *	1.58	0.128
	*p* ^1^	0.042 *			<0.001 *	0.034 *						

* *p* < 0.05, *p*^1^: Friedman Test, *p*^2^: Mann–Whitney U Test.

**Table 3:** Shoulder Range of Motion Levels.

Shoulder ROM		Home Exercise Group	Manual Therapy Group	Tele Rehabilitation Group	Between Groups					
					HE-MT		HE- TR		MT-TR	
		X ± SD	X ± SD	X ± SD	z	*p* ^2^	z	*p* ^2^	z	*p* ^2^
Flexion	Before Treatment	170.35 ± 4.74	173.25 ± 2.45	171.50 ± 4.89						
	After Treatment 8th week	173.55 ± 3.12	179.5 ± 1.54	178 ± 2.51	3.08	<0.001 *	2.96	0.01 *	1.52	0.143
	Follow-up 12th week	174.75 ± 1.97	179.00 ± 2.62	180.0 ± 0.0	2.59	<0.001 *	3.29	<0.001 *	1.93	0.058
	*p* ^1^	0.028 *	<0.001 *	<0.001 *						
Abduction	Before Treatment	169.25 ± 5.2	172.60 ± 3.76	169.75 ± 4.72						
	After Treatment 8th week	172.0 ± 4.70	179.5 ± 1.54	177.5 ± 2.56	3.53	<0.001 *	3.41	<0.001 *	1.51	0.159
	Follow-up 12th week	173.25 ± 3.73	179.5 ± 1.54	179.0 ± 2.62	3.48	<0.001 *	3.45	<0.001 *	1.60	0.112
	*p* ^1^	0.026 *	<0.001 *	<0.001 *						
İnternal Rotation	Before Treatment	79.75 ± 6.97	81.5 ± 5.16	81.25 ± 5.59						
	After Treatment 8th week	82.45 ± 5.91	89.0 ± 2.05	86.75 ± 2.94	3.13	<0.001 *	2.93	<0.001 *	1.48	0.162
	Follow-up 12th week	84.2 ± 5.31	89.25 ± 1.83	89.75 ± 1.12	3.18	<0.001 *	3.19	<0.001 *	1.68	0.104
	*p* ^1^	<0.001 *	<0.001 *	<0.001 *						
External Rotation	Before Treatment	87.25 ± 3.43	88.75 ± 2.75	87.50 ± 3.44						
	After Treatment 8th week	87.9 ± 3.37	90.0 ± 0.0	89.25 ± 2.45	3.01	<0.001 *	2.88	<0.001 *	1.55	0.135
	Follow-up 12th week	88.25 ± 2.94	90.0 ± 0.0	89.5 ± 1.54	2.35	<0.001 *	2.31	0.027 *	0.99	0.334
	*p* ^1^	0.223	0.258	0.394						

* *p* < 0.05, *p*^1^: Friedman Test, *p*^2^: Mann–Whitney U Test.

**Table 4:** Q-DASH Levels.

Q-DASH	Home Exercise Group	Manual Therapy Group	Tele Rehabilitation Group	Between Groups					
				HE-MT		HE-TR		MT-TR	
	X ± SD	X ± SD	X ± SD	Z	*p* ^2^	z	*p* ^2^	z	*p* ^2^
Before Treatment	46.36 ± 7.16	51.7 ± 8.94	51.14 ± 5.74						
After Treatment 8th week	26.48 ± 9.07	18.3 ± 12.01	30.11 ± 7.83	4.65	<0.001 *	1.401	0.128	5.21	<0.001 *
Follow-up 12th week	20.45 ± 6.43	6.71 ± 9.31	18.98 ± 4.68	5.23	<0.001 *	0.550	0.663	5.03	<0.001 *
*p* ^1^	<0.001 *	<0.001 *	<0.001 *						

* *p* < 0.05, *p*^1^: Friedman Test, *p*^2^: Mann–Whitney U Test.

**Table 5:** Patient Satisfaction VAS Levels.

	Home Exercise Group X ± SS	Manual Therapy Group X ± SS	Tele Rehabilitation Group X ± SS	Between Groups					
				HE-MT		HE-TR		MT-TR	
				z	*p*	z	*p*	z	*p*
Patient SatisfactionVAS Follow-up 12th week	6.10 ± 0.72	7.70 ± 0.47	7.90 ± 0.45	4.24	<0.001 *	4.03	<0.001 *	1.02	0.335

* *p* < 0.05.

## Data Availability

Data are contained within the article.
